# Cephradine-Induced Toxic Epidermal Necrolysis: A Case Report of Treatment With Etanercept

**DOI:** 10.7759/cureus.32657

**Published:** 2022-12-18

**Authors:** Rawan Hubail, Layal Alnajjar, Ameen Al Awadhi

**Affiliations:** 1 Dermatology, Salmaniya Medical Complex, Manama, BHR

**Keywords:** scorten, cephradine, enbrel, etanercept, ten, toxic epidermal necrolysis

## Abstract

Toxic epidermal necrolysis (TEN) is a rare yet life-threatening severe cutaneous adverse reaction (SCAR) to various causative agents, including medications, vaccinations, infections, and malignancies, in addition to some other uncommon external stimuli. TEN is characterized by the sudden appearance of generalizeddusky erythematous targetoid macules with a purpuric center, which coalesces to form bullae and flaccid blisters, leading to an eventual sheet-like epidermal detachment of all necrotic areas. Extensive epidermal denudation in TEN is usually accompanied by thermoregulatory impairment, insensible fluid loss, and hemodynamic instability. The severity of presentation for TEN is calculated through the use of a "Severity-of-Illness Score for Toxic Epidermal Necrolysis" (SCORTEN) score.

Certain medications, including antibiotics, anticonvulsants, corticosteroids, and nonsteroidal anti-inflammatory drugs, are considered the primary cause of this dermatosis. In this report, we describe a case of TEN caused by Cephradine, a first-generation cephalosporin antibiotic drug, in a 43-year-old South Asian male who presented to the emergency department one day after receiving Cephradine for the treatment of cellulitis. At presentation, this patient had a calculated SCORTEN score of 4 according to the SCORTEN criteria found in the literature, placing his mortality rate at 58%. His treatment plan consisted of a single 50mg dose of Etanercept (ENBREL), a soluble anti-tumor necrosis factor alpha inhibitor (TNF-α) monoclonal antibody, as an emergent intervention at presentation, along with cyclosporine and hydrocortisone in tapered doses. This is the first reported case of severe TEN in the Kingdom of Bahrain successfully treated with a TNF-α inhibitor, Etanercept in this case, achieving complete healing and remission within 20 days of presentation, after initially showing a poor prognosis and a high risk of fatality.

## Introduction

Toxic epidermal necrolysis (TEN), also known as Lyell's syndrome, is a severe and possibly fatal mucocutaneous condition first described by Lyell in 1956 as a delayed-type hypersensitivity reaction characterized by the sudden dispersal of dusky red or violaceous necrotic macules over the body followed by generalized cutaneous denudation as a result of the introduction of a causative agent [1]. Mortality rates in TEN cases range from 30% to 70% depending on the total body surface area (TBSA) affected and the SCORTEN score calculated for each case at the time of onset [2]. Also, apoptosis-induced keratinocyte death results in extensive epithelial and mucous membrane depletion in TEN [3].

Cutaneous involvement in TEN may vary from partial to full-thickness apoptosis depending on the severity of widespread cutaneous necrosis, causing severe detachment of the cutaneous layer at the dermal-epidermal junction. Also, mucosal membrane involvement in TEN is an important indicator of the severity of the disease. Similarly, some probable ophthalmic changes that may present in TEN cases include periorbital edema, entropion, corneal erosions, and sloughing of the skin on and around the eyelids. Likewise, airway compromise, dyspnea, and hypoxemia due to compressing edema and bronchial epithelial sloughing are also seen in severe presentations. Urogenital involvement in TEN is mainly present in severe disease and may include erosive and ulcerative vaginitis, vulvar bullae, and vaginal synechiae in females, as well as scrotal erosions, urethritis, phimosis, and urethral meatal stenosis in males, with occasional long-term sequelae [3].

Several treatment modalities have been explored in literature for the treatment of TEN, however, no definitive standard of treatment has yet been established for the management of TEN since each case is distinctive in presentation and severity. Although Etanercept, cyclosporine, high-dose systemic corticosteroids, and, more recently, intravenous immunoglobulins and plasmapheresis have been shown to slow disease progression, their efficacy remains unknown [4,5].

TEN is thought to be caused by keratinocyte apoptosis, which ultimately leads to full-thickness epidermal necrosis. TNF-α is thought to be involved in the disease's pathology cascade [3]; thus, Etanercept, a TNF-α antagonist, is thought to be effective in treating TEN due to its pathology cascade mediating effects [6]. Based on the literature, patients with TEN usually stay in the hospital and receive treatment for an average of 25 days [7]. Here, we present the case of a middle-aged South Asian male who developed TEN for the first time after taking the Cephradine antibiotic. In this case, after initiating treatment with a single dose of the TNF-α blocking drug Etanercept upon presentation, no newly emerging apoptotic lesions were noted as of the second post-administration, and epidermal detachment seized after five days of presentation. Ten days later, the process of re-epithelialization was underway over the areas of sloughed skin. By the 20th day of hospitalization (the day of discharge), complete healing of all patches was noted. In comparison to earlier studies in the literature, this patient was deemed entirely disease-free after only three weeks of follow-up [3,8].

## Case presentation

**Figure 1:**
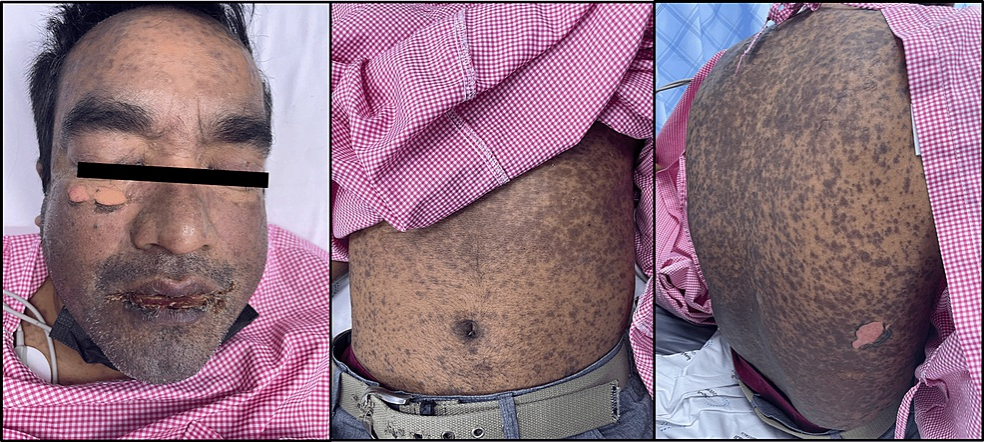
Upon presentation to the ED: Eruptions of painful apoptotic macular exanthema all over the body with evident mucosal involvement.

A 43-year-old South Asian male with a history of hypothyroidism and gouty arthritis presented to the emergency department with extensive, generalized, apoptotic, and dusky confluent lesions resembling atypical targetoid macules with a central purpuric hue, along with areas of blistering and flaccid bullae mostly noted on the face, upper limbs, trunk, and back. Small areas of sloughed skin and vesiculobullous patches were noted in the skin folds of the neck and axilla. Nikolsky's sign was positive at the time of the presentation. These cutaneous lesions covered around 50% of the patient’s total body surface area (TBSA). Mucosal membrane involvement was also observed in the form of perioral edema, crusting and erosions, glossitis, and angular cheilitis with mild bleeding. Similarly, ocular involvement was also noted in this patient through the presence of periorbital edema, ptosis of the eyelids, bilateral conjunctivitis and ocular congestion, corneal erosions, and sloughing of the skin on and around the eyelids, with complete detachment of the eyelashes (Figure 1). The patient was in immense pain and discomfort and therefore avoided all unnecessary movement or speech. The patient also displayed signs of shortness of breath and discomfort upon swallowing with mild regular tachycardia and a fever reaching 39.8°C.

Based on this patient's medical history, these cutaneous changes appeared one day after taking a single dose of Cephradine, a first-generation cephalosporin antibiotic that was prescribed in private practice for the treatment of minor lower-limb cellulitis. Other than taking this antibiotic, there were no other significant changes in the patient's daily routine within the week before his presentation. The patient denied having any known allergies or any significant family history. He is a healthy, non-smoker and a non-alcoholic adult and has denied any illicit drug use. There was no history of animal exposure, insect bites, or recent travel. All the clinical findings observed, along with the patient’s medical history, are consistent with the diagnosis of TEN, presenting as a reactionary response to Cephradine antibiotic administration.

Following this diagnosis, a SCORTEN score was used to quantify the severity and determine the prognosis of the case [9]. The total score for this patient was calculated by the presence of four of the listed risk factor criteria introduced by Parker et al., including the patient's age (43 years), the TBSA affected (above 50%), elevated serum glucose levels, and high serum urea levels at the onset. These results highlight the gravity of this presentation and place the patient’s SCORTEN score and risk of mortality at 58% upon onset.

Prompt multidisciplinary array of assessments were done to properly evaluate this patient's condition and establish an effective treatment plan, including assessments from the Ear, Nose, and Throat (ENT) department, the Ophthalmology department, the Internal medicine department, and the Intensive Care Unit (ICU) team. An extensive evaluation of this patient's initial lab results also showed a low white blood cell count, markedly low calcium and magnesium levels, borderline potassium levels, high serum amylase, and elevated cardiac enzymes. Therefore, electrolyte and fluid replacement were established early and aggressively.

Based on the severity of this presentation, the patient was admitted to the ICU for the first two days of his stay until effectively stabilized, then was transferred to the burn unit isolation room for the remainder of his hospitalization period in order to maintain a sterile environment and avoid secondary infections, sustain proper thermoregulation, provide appropriate supportive care, wound care, and specialist nursing, as well as to maintain close patient observation, and monitor for possible complications and sequelae of acute skin failure.

Upon presentation to the resuscitation room in the emergency department, a single dose of Etanercept, a TNF-α inhibitor, was given as an emergent treatment via subcutaneous injection, along with high doses of intravenous cyclosporine and hydrocortisone. Testing for Purified Protein Derivatives (PPD) or QuantiFERON-TB Gold blood test prior to the administration of Etanercept was not possible in this case since epidermal denudation has already commenced and associated complications make it challenging to delay treatment and wait for the results of these tests which need over 24 to 36 hours to be reported. This patient also received Cyclosporine for a total of 19 days, beginning with a dose of 3mg/kg/day in two divided doses daily for the first two days of admission, followed by a higher dose of 5mg/kg/day in two divided doses daily for the next three days. From the seventh day on, the dose was gradually tapered down to the minimum until it was discontinued on the nineteenth day of admission. The patient was also given moderate doses of hydrocortisone for the first six days of his admission, with a dose of 3mg/kg/day administered intravenously every eight hours. The doses of hydrocortisone were gradually reduced and switched to oral prednisolone upon discharge. After discharge, the patient was given a follow-up appointment after three weeks at the dermatology clinic and ophthalmology clinic in order to track his healing progress and perform a PPD test.

During hospitalization, wound care for this patient consisted of a body bath using saline and chlorhexidine solution performed every other day, followed by the application of fusidic acid antibiotic cream all over the body, complemented with a layer of emollients and covered with a sterile non-adhesive gauze dressing. Saline application every two hours is vital. Also, the patient's oral cavity was rinsed with a sodium bicarbonate mouthwash three times daily, along with frequent saline rinses and white petroleum jelly application on the lips. As re-epithelialization and healing were achieved, the use of fusidic acid cream and the non-adhesive dressing were discontinued, and the patient’s body was just cleansed and smeared with oil four times daily and kept open without cover until discharge. Other supportive measures were also taken such as the insertion of a nasal cannula, nasogastric tube, and a foley catheter since the patient was nonambulatory for most of his hospitalization. Appropriate calculation of parenteral nutrition and oxygen saturation upkeep is very important during the management of a patient with TEN.

Throughout the hospitalization period, a series of peripheral blood, wound, skin, and perianal cultures were done. All cultures were sterile on the first day of admission, with the exception of the perianal culture, which returned a positive result for Extended-Spectrum Beta-Lactamase (ESBL), where the patient was given a seven-day course of Piperacillin Tazobactam antibiotics in addition to his other prescribed drugs. A repeated wound culture revealed a Methicillin-Resistant Staphylococcus Aureus (MRSA) hospital-acquired infection on the sixth day of stay, therefore the patient was also started on vancomycin coverage until the day of discharge. Four days prior to discharge, the repeated wound culture showed a growth of Klebsiella ESBL, but since vancomycin was already being administered, no additional treatment was necessary. All cultures displayed a negative result for any growth upon discharge.

**Figure 2:**
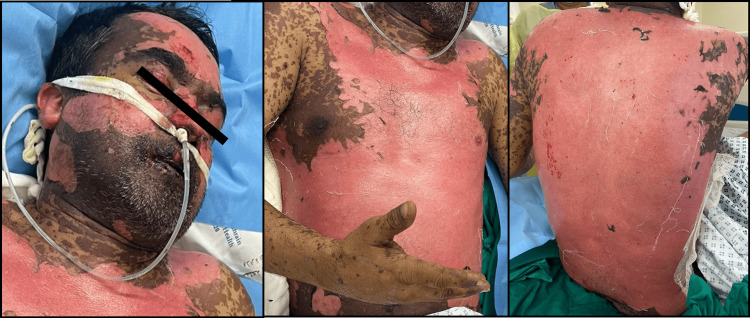
Seventh day of hospitalization: Complete sheet-like epidermal detachment of all the cutaneous necrotic areas.

**Figure 3:**
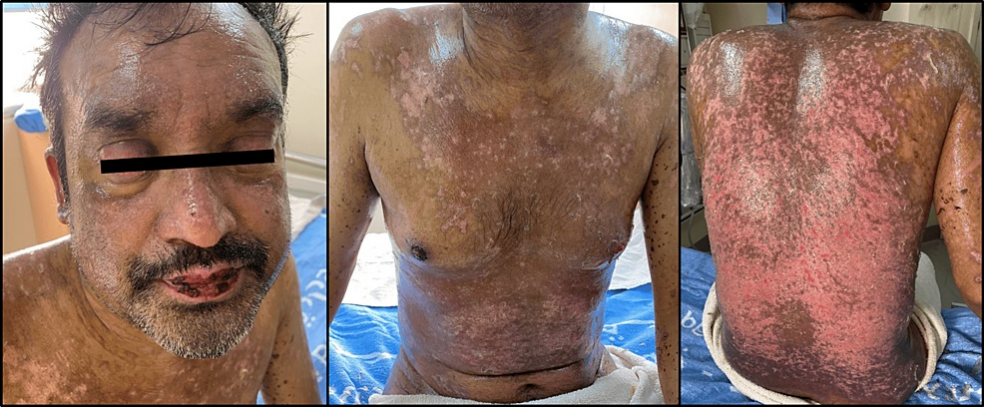
14th day of hospitalization: re-epithelialization and healing of the cutaneous layers.

**Figure 4:**
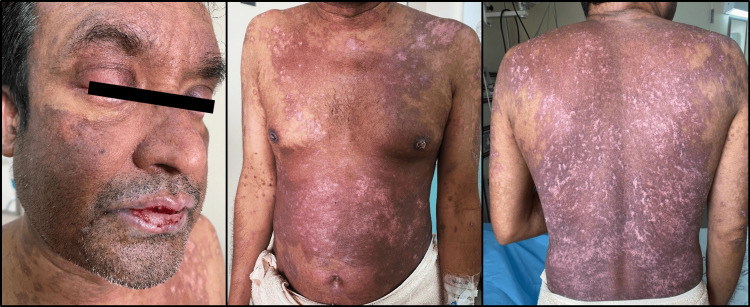
20th day of hospitalization upon time of discharge: substantial healing and resurfacing of the cutaneous layers.

**Figure 5:**
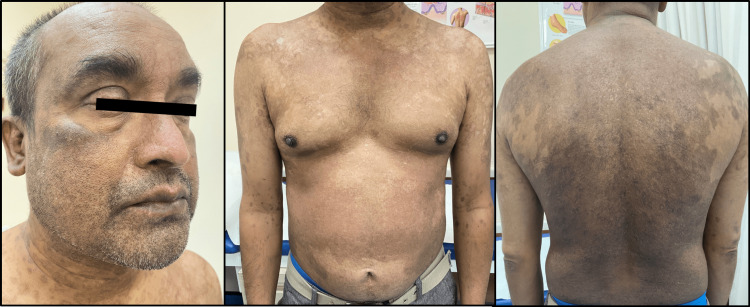
During the outpatient follow-up visit after three weeks of discharge from the hospital: Complete remission of disease with some hypo- and hyper-pigmented areas post-healing.

The patient's progress was documented with serial photographs taken at seven-day intervals until discharge and at his follow-up visit post-discharge. Figure 2 depicts the extent and severity of epidermal detachment on the seventh day of hospitalization. Figure 3 marks the beginning of skin healing and re-epithelialization 14 days after admission. Figure 4 presents the final stages of skin healing, mucus membrane resurfacing, ocular improvement, and overall clinical progress prior to discharge. Finally, Figure 5 displays complete cutaneous healing and remission three weeks after discharge.

## Discussion

TEN is an acute and severe mucocutaneous adverse drug reaction caused by certain pharmaceutical drugs. Although cephalosporin-related dermatoses are extremely rare, they have previously been reported [10]. It causes a rapid, widespread reactionary mucocutaneous response, which usually presents three days after the offending drug is introduced [8]. This process has been linked to the presence of several apoptotic pathways and cell-mediated cytotoxic hypersensitivity drug reactions. Female predominance in drug-induced TEN was demonstrated by Zhang et al. (2019) and other researchers in the literature [11,12], however, the male patient was represented in this reported case study.

Pharmacological interventions for TEN include anti-TNF-α inhibitors, corticosteroids, cyclosporine, IVIG, and plasmapheresis, according to the literature. Therapeutic modalities should be promptly introduced within the first 48-hour window from the onset of symptoms in any case of TEN to initiate rapid cessation of disease progression and prevent a poor prognosis or fatality. A novel approach to treat this condition is through interrupting the apoptotic pathways and activating CD8+ cytotoxic lymphocytes and natural killer (NK) cells [13]. The TNF-α pathway is blocked by the monoclonal antibody Infliximab or the soluble TNF-α fusion protein Etanercept, which reduces mortality, halts disease progression, and accelerates re-epithelialization and healing time [13,14]. Etanercept is currently considered to be the most effective anti-TNF-α agent for SJS (Steven Johnson Syndrome) and TEN patients. In our case, employing a single dose of 50mg Etanercept subcutaneously at the time of onset has resulted in a vast decrease in the mortality rate, a rapid halting of disease progression as well as a faster rate of re-epithelialization. The SCORTEN score for this patient upon presentation was 4, depicting a possible mortality rate of 58%. Upon completion of inpatient care, the patient demonstrated complete cutaneous re-epithelialization and major clinical improvement, eliminating the risk of a fatality and demonstrating the efficacy and safety of the use of Etanercept as a combination therapy with standard treatments in the management of SJS/TEN. The use of a single dose of Etanercept is also supported in some cases within the literature, but the evidence is limited [6,15].

Moreover, TEN is associated with delayed-type hypersensitivity reactions. Methylprednisolone was another treatment used in conjunction with Etanercept in this case. Methylprednisolone downregulates pro-inflammatory cytokines. Traditional corticosteroids are therefore widely used in the treatment of SJS/TEN due to their immunosuppressive properties. However, adverse events associated with high-dose administration limit their long-term use. Within recent literature, the use of Etanercept or combination therapy is considered superior to the use of traditional steroids alone. Wang et al. suggested that the SCROTEN-based predicted mortality rates were decreased by 17.7% with Etanercept and 8.3% with prednisolone [15], hence, the use of Etanercept or combination therapy is suggested to be more effective.

In addition, tapered doses of Cyclosporine were used in conjunction with Etanercept and Methylprednisolone to treat this case. Cyclosporine is a calcineurin inhibitor and a potent immunomodulator. Studies revealed rapid re-epithelialization within an average of 12 days, with the use of cyclosporine. According to several case reports, patients were stabilized and blistering was successfully stopped within 24 hours of starting cyclosporine, with no adverse events reported. It inhibits the production of cytokines (most notably Interleukin-2), which play a role in the regulation of T-cell proliferation. Unfortunately, 20% of deteriorating cases of TEN have a high tendency of developing acute renal failure, limiting the prolonged use of cyclosporine as it aids in increasing the rate of renal failure [15].

The use of a combination triple-therapy (Etanercept, Methylprednisolone, and Cyclosporine) in this severe case of TEN has greatly aided in promptly ceasing the disease progression, promoting rapid re-epithelialization of sloughed epidermal areas, and improving the patient’s overall clinical condition, eliminating the risk of a fatal prognosis. With that said, the most crucial steps in the management of any TEN cases are early diagnosis, rapid elimination of the offending agent, emergent hemodynamic stabilization, maintaining airway potency, and continuous supportive care in a sterile setting.

This case presentation of a cephalosporin-induced TEN may aid clinicians in better understanding the disease presentation and progression, ways of early detection, relevant scoring criteria, and treatment options for this disease, including the possible early introduction of Etanercept as a treatment modality of choice or as a part of combination therapy.

## Conclusions

To summarize, TEN is a devastating and emergent adverse drug reaction, with a very high fatality rate, characterized by generalized acute mucocutaneous apoptotic macules and sheet-like sloughing of the skin caused by the administration of an offending drug. The prompt elimination of the offending substance, the development of a proper SCORTEN score, the urgent initiation of pharmacological treatment, and the continuous supportive and specialist care provided by an ICU and/or burn unit are the most crucial aspects in managing TEN.

This is the first case in the Kingdom of Bahrain where Etanercept, a TNF-α antagonist, has been administered as part of the treatment plan in addition to cyclosporine and corticosteroids for the management of severe TEN, and which has presented a very successful outcome. We conclude that the addition of Etanercept to the treatment regimen has significantly contributed to this case's prompt resolution and prognosis. With that said, one limitation to this notion is that it is hard to ascertain how much Etanercept has helped in this case, given that the patient also received a combination therapy of moderate to high doses of cyclosporine and hydrocortisone. Our findings may aid clinicians in better understanding and treating drug-induced TEN in the future with the use of Etanercept as an adjunctive treatment as opposed to traditional pharmacological treatments alone, however, a further clinical and mechanistic study on a case-by-case basis is required before making an educated decision regarding the proper management plan of each distinct TEN presentation.
